# Mortality profiles in a country facing epidemiological transition: An analysis of registered data

**DOI:** 10.1186/1471-2458-9-47

**Published:** 2009-02-02

**Authors:** Luis Huicho, Miguel Trelles, Fernando Gonzales, Walter Mendoza, Jaime Miranda

**Affiliations:** 1 Department of Paediatrics, Universidad Nacional Mayor de San Marcos, Lima, Peru; 2 Department of Paediatrics, Instituto Nacional de Salud del Niño, Lima, Peru; 3 School of Medicine, Universidad Peruana Cayetano Heredia, Lima, Peru; 4 Pan American Health Organization, Lima, Peru; 5 United Nations Population Fund, Lima, Peru; 6 Department of Epidemiology and Population Health, London School of Hygiene and Tropical Medicine, London, UK; 7 School of Public Health and Administration, Universidad Peruana Cayetano Heredia, Lima, Peru

## Abstract

**Background:**

Sub-national analyses of causes of death and time-trends help to define public health policy priorities. They are particularly important in countries undergoing epidemiological transition like Peru. There are no studies exploring Peruvian national and regional characteristics of such epidemiological transition. We aimed to describe Peru's national and regional mortality profiles between 1996 and 2000.

**Methods:**

Registered mortality data for the study period were corrected for under-registration following standardized methods. Main causes of death by age group and by geographical region were determined. Departmental mortality profiles were constructed to evaluate mortality transition, using 1996 data as baseline. Annual cumulative slopes for the period 1996–2000 were estimated for each department and region.

**Results:**

For the study period non-communicable diseases explained more than half of all causes of death, communicable diseases more than one third, and injuries 10.8% of all deaths. Lima accounted for 32% of total population and 20% of total deaths. The Andean region, with 38% of Peru's population, accounted for half of all country deaths. Departmental mortality predominance shifted from communicable diseases in 1996 towards non-communicable diseases and injuries in 2000. Maternal and perinatal conditions, and nutritional deficiencies and nutritional anaemia declined markedly in all departments and regions. Infectious diseases decreased in all regions except Lima. In all regions acute respiratory infections are a leading cause of death, but their proportion ranged from 9.3% in Lima and Callao to 15.3% in the Andean region. Tuberculosis and injuries ranked high in Lima and the Andean region.

**Conclusion:**

Peruvian mortality shows a double burden of communicable and non-communicable, with increasing importance of non-communicable diseases and injuries. This challenges national and sub-national health system performance and policy making.

## Background

Worldwide improvements in mortality over the last 50 years have not been uniform for all countries. A pattern of divergence in life expectancy at birth has been reported globally in recent times despite initial signs of convergence, thus indicating reversal in the progress towards reduction of mortality differences between populations [1].

Global mortality estimates have been used to assess the burden of disease and risk factors at global and regional levels [2,3]. This information has been widely used as a tool for policy analysis and policy decision-making by many international agencies and research groups [4], and also in many countries. However, the use of global or regional estimates to inform local decisions has limitations. Morbidity and mortality patterns are heterogeneous within different regions of the world [5] but also, and most importantly, within countries [6]. Hence, there is the need to strengthen the quality, availability, analysis, and use of local data and statistics to meet country as well as international needs [7]. This approach can be helpful at two levels in public health and policy-making. Firstly, it can result in a better resource allocation to those areas with greatest needs. Secondly, it can help to identify those areas that can benefit most from appropriate preventive intervention programmes.

It is becoming widely accepted that non-communicable or chronic diseases are now major causes of death and disability in low – and middle-income countries [8-10]. However, societies are not static, and many countries are experiencing different stages of transition: demographic [11], epidemiological [12], nutritional [13,14], or health [15] transitions. These changing processes and trends underlie the importance of addressing local health situations in a systematic way based on locally available data, disaggregated by relevant factors such as socioeconomic status, ethnicity and geographic region, to inform local policy making. In this regard, progress is being made in the description of local patterns of transition for developing countries, including China and India in Asia [16-18], as well as Chile, Guatemala, Mexico and Uruguay in Latin America [19-21].

The interest on Latin America's epidemiological transition is not new. The seminal work of Frenk et al. in the early 1990's had already raised the issue [22]. They described some unique features of the process in Latin American countries. In Peru the topic has also been gaining momentum in the last few years. However, such approaches have only included so far a patchy insight of the epidemiological transition in a specific city in Peru's interior which dealt with the clinical implications of the process from a research and services perspective [23], and a description of the stages of transition at country-level, trying to apply lessons from historical trends in order to re-think the overall Peruvian health system [24]. The latter study describes several features of a protracted epidemiological polarization in a transitional country like Peru. One of such features is the unequal distribution of wealth and health status within the country [24]. Also, the authors highlight striking contrasts for several important health indicators, including the mortality rate due to communicable diseases, cardiovascular diseases, and neoplasms, between Lima, the richest department of the country, and Huancavelica, the poorest one [24]. However, the study did not perform an in-depth analysis of sub-national causes of death. To the best of our knowledge, there are no previous studies aimed at assessing national and sub-national mortality patterns in Peru by using available data from the vital registration system.

National and within-country regional analyses of causes of death as well as time-trends of such patterns are, therefore, of vital importance to identify priorities for public health interventions, to guide selection of health interventions for implementation, to evaluate their impact on health, and to increase their cost-effectiveness by identifying the most appropriate target groups. The aim of this paper is to describe Peru's national and regional mortality profiles from all causes and for all ages between 1996 and 2000, using registered vital statistics and methods corrected for under-registration.

## Methods

### Context

Peru, a middle-income country according to World Bank's definition [25], has a population of 27 million inhabitants, with 7.5 million people living in the capital city, Lima [26]. Gross national income per capita was $2,650 in 2006 [27], and life expectancy at birth was 69 years for males and 73 years for females for the year 2004 [28].

Peru is divided politically in 24 departments and one constitutional province, Callao. Peru is also usually divided in three different regions, namely the coast, the Andes or Sierra, and the rainforest. Each region has distinct geographic, cultural and socioeconomic characteristics. In 2000, human development index ranged from 0.46 in Apurimac (an Andean department) to 0.75 in Lima and Callao (coastal cities) [29]. Proportions of families living below the poverty line by department vary also dramatically [30]. A substantial proportion of Andean populations have still Quechua or Aymara as their native language. Communities living in rainforest areas, particularly those in most remote areas, speak a variety of local dialects. Not fluency in Spanish, the official national language, along with poverty and with particular health conceptions that contrast with western medicine philosophy, limits dramatically the access of these groups to good quality health services and other basic needs. Thus not surprisingly, Peru's national health statistics as country averages hides great disparities between regions and departments and even within them [31].

As for existence and functionality of a vital registration system, Peru has been included as a country with low quality information on causes of death, based on registered data reported by member states to the World Health Organization for 1990 or later [32]. This underscores the need to transparently assess the pitfalls and limitations of the country – and local-level registered mortality data, and also of any correction method used.

### Mortality and cause-of-death estimates

The methodological approach to estimate mortality levels and trends is based on a previous paper dealing with under-five mortality profiles in Peru, whose rationale and basics are described in further detail elsewhere [33]. Registered mortality data, provided by the Peruvian Ministry of Health for the period 1996 through 2000, were corrected for under-registration following a method developed by the Centro Latino-Americano de Demografía (CELADE, Latin American Centre for Demography) and adopted by the Pan American Health Organization (PAHO) [34]. The causes of death in these registered mortality data were based on clinical diagnosis prior to death and on medical autopsy. The CELADE method is described in detail in the technical notes appendix of PAHO's Health Statistics from the Americas report [34]. In brief, a CELADE model life-table for Peru that fitted the age and sex distribution of the population and of registered deaths for each year between 1996 and 2000 was identified. The mortality rates corresponding to these model life-tables were then assigned to each department for each particular year, that is, the redistribution of unregistered deaths into different age – and sex groups and different causes of death was done for each department separately. The registered mortality data was also adjusted for deaths unknown by age and sex. Then the number of deaths unknown by age was redistributed into known age groups by multiplying the number of deaths for each sex and age group by an adjustment factor. This factor is obtained from the total number of deaths divided by the number of deaths stated by age. For rate calculations various assumptions were made about the registered mortality data. First, that all deaths coded to an external cause were in fact due to an external cause and that none of deaths coded to other cause categories, including ill defined conditions, were in fact due to external causes. Second, that the distribution of the unregistered deaths into cause categories by age group and sex was the same of that among registered deaths. Thus unregistered deaths were redistributed into corresponding cause categories by age and sex in the same proportions as the registered deaths. Third, the estimated age and sex rates were calculated by accumulating the registered and unregistered deaths in a given period, by cause category and dividing by the sum of the corresponding estimated populations. The infant mortality rate was calculated using the estimated number of live births or the estimated population under 1 year of age. Accordingly, the reference life-tables were applied to correct the under-registration of mortality in the corresponding department.

These results were compiled by PAHO and expressed as deaths per 100,000 population [34]. Coding of mortality causes was performed using ICD-9 from 1996 to 1998, and ICD-10 from 1999 onwards. In order to match ICD-9 and ICD-10 classification systems, classification of cause of death was made according to PAHO recommendations [35].

The method to correct for under-registration assumes that for each age group and sex there is an expected number of deaths, which results from multiplying the population by the central mortality rate (_n_m_x_) of the life-table for the 1995–2000 period [34,36,37]. A value for expected deaths is obtained which, as anticipated, outnumbers those recorded. The difference between calculated expected and recorded deaths becomes the under-registration gap for each specific age group and sex. This expected versus recorded difference in deaths is then redistributed for each sex and age group, as recommended by the PAHO approach.

For estimating age-standardized number of deaths in each department we used the direct standardization in which the age-specific rates for the population of interest are applied to a standard population.

### Data Analysis

Total deaths were calculated for each department. Mortality data for all causes were calculated for all age groups and for both sexes. For the purposes of this paper four regions were considered: Lima, coastal, Andean and rainforest regions [38]. Due to its population size, Lima and Callao were grouped into a single region, Lima.

Four main analyses were carried out. First, the main causes of death at national level and by age groups for the period 1996 through 2000 were determined. Average values of mortality rate for a given cause of death for the five-year study period were calculated and are presented as percentages for each cause. In order to calculate main causes of death by age groups, expressed as mortality rates per 100,000 population for the same period, total mortality due to an individual cause of death in a specific age group was divided by the total population in the same age group.

Second, the main causes of death by geographical regions for the aggregated period 1996 through 2000 were calculated. The number of causes included in this study is 64. This resulted from the fact that we combined ICD-9 (61 causes) and ICD-10 (67 causes), which have some differences. Thus syphilis was present in ICD-9 but not in ICD-10, while uterine malignancies appeared disaggregated in ICD-10. Diseases of pulmonary circulation and other forms of heart disease were disaggregated in ICD-10. As for neonatal causes, hemolytic disease disappeared and bacterial sepsis was included in ICD-10. Finally, in the injuries category, accidents caused by machinery and by cutting and piercing instruments were removed in ICD-10, and accidental threats to breathing exposure and to electric current were added in ICD-10.

Third, the transition of mortality profiles between three broad groups of causes of mortality, similar to those recently used in some international reports [10,39], was assessed by each department, setting arbitrary thresholds for the different causes of death, as previously reported [33]. The major groups [10,39] used for this analysis included: a) Communicable diseases that encompass maternal and perinatal conditions, and nutritional deficiencies; b) Non-communicable diseases which included cardiovascular diseases, diabetes mellitus, cancer and chronic respiratory diseases; and, c) Injuries. In 1996, Peru had 40%, 49.8% and 10.2% of deaths due to communicable diseases, non-communicable diseases, and injuries, respectively. The 1996 values of national averages of causes of death were used to establish baseline reference thresholds. These cut-offs allowed the evaluation of any change in pattern of causes of death by departments, from 1996 to 2000. That is, if a given department had more than 40% of deaths due to communicable diseases, then it was considered as having a profile 1. If it had more than 49.8% of non-communicable deaths and less than 40% of communicable deaths, it was categorized as having a profile 2. Profile 3 was defined as any department with more than 10.2% deaths due to injuries and less than the 1996 percentage for communicable and non-communicable causes. Mortality rate, total population, and proportion of deaths for the departments included in each profile group were also calculated.

Fourth, the evolution of mortality time-trends was evaluated for each department and region. Cumulative reduction in mortality rate in the 1996–2000 period was estimated by calculating the annual reduction slope for each department through fitting a regression line of mortality rate levels on calendar years; the five-year cumulative reduction was obtained from the annual slopes. For illustrative purposes in this particular analysis, nine groups of death causes were used to inform the patterns of change for each area, rather than the three-groups used before. The following nine groups were used: infectious diseases, maternal and perinatal conditions, nutritional deficiencies and nutritional anaemia, circulatory diseases (excluding those of infectious origin), cancer, chronic airway diseases, diabetes mellitus, and other chronic diseases and injuries.

Data were analyzed using SPSS, version 12.0. Graphs and tables were constructed to show the distribution of deaths. Deaths are expressed as percentages when disaggregating deaths by causes, or as rates per 100,000 when evaluating total national and regional mortality averages.

## Results

The uncorrected data showed in average 88,967 deaths for the period 1996–2000. The average proportion of deaths of unknown age for the same period was 1.1%, and the average proportion of deaths assigned to the category 'symptoms, signs and ill-defined conditions' was 18.6%. Number and proportion of deaths assigned to this category by age are shown as an appendix (see Additional file 1). Under-five mortality rate obtained from the model life table was reported in detail elsewhere [33]. Summary life table measures such as life expectancy and mortality risk by age are also shown as an appendix (see Additional file 2).

The average number of deaths for the period 1996–2000 was 168,346. The total number of deaths for the study period was 841,732. During this period, Lima, coastal region other than Lima, Andean and rainforest regions accounted for 20.1%, 20.1%, 50.0% and 9.7% of deaths, respectively, while they represented 31.7%, 21.0%, 37.6% and 9.7% of country's population.

### 1. Main causes of death at national level and most common causes of death by age groups

At national level, in 1996 there were 67,245 (40.0%), 83,669 (49.8%) and 17,227 (10.2%) deaths attributed to the groups of communicable diseases, non-communicable diseases, and injuries, respectively. The corresponding figures for the same groups in year 2000 were 56,752 (33.6%), 93,207 (55.1%) and 19,159 (11.3%).

Table 1 shows under-registration by department and by year from 1996 through 2000, and proportion of families living below the poverty line. Standardized death rate and total population by department and by year are shown as an appendix (see Additional file 3)

**Table 1:** Mortality under-registration by year (period 1996–2000), and proportion of families living below poverty line (2001).

	**1996**	**1997**	**1998**	**1999**	**2000**	
	**Mortality under-registration (%)**					Families living below poverty line (%)*
**Coast**	**29.4**	**27.8**	**25.0**	**33.0**	**31.2**	**43.1**
Lima and Callao	19.4	20.6	18.5	23.8	21.2	33.4
Ica	26.7	29.7	21.9	32.7	32.1	41.7
La Libertad	40.6	41.2	28.9	39.9	40.7	52.1
Lambayeque	38.9	42.2	26.7	47.3	44.7	63.0
Moquegua	44.2	39.9	41.5	43.2	44.5	29.6
Piura	61.7	44.2	51.7	62.8	60.1	63.3
Tacna	28.3	26.7	24.8	29.9	34.1	32.8
Tumbes	48.3	1.1	36.5	47.2	52.4	46.8
**Andes**	**49.0**	**54.0**	**54.1**	**53.4**	**57.8**	**69.3**
Ancash	64.2	90.4	57.1	59.2	64.2	61.1
Apurimac	51.1	72.3	80.9	59.9	68.9	78.0
Arequipa	30.7	36.0	34.5	39.8	48.1	44.1
Ayacucho	76.1	74.4	78.5	76.3	75.3	72.5
Cajamarca	64.2	69.8	70.9	72.7	69.1	77.4
Cusco	38.2	41.5	44.3	44.3	51.1	75.3
Huancavelica	73.8	76.7	73.3	75.2	74.9	88.0
Huanuco	50.2	39.4	41.7	45.5	49.6	78.9
Junin	37.3	43.8	39.8	42.4	43.1	57.5
Pasco	51.5	8.9	52.8	54.8	57.3	66.1
Puno	32.7	36.4	52.8	42.0	55.8	78.0
**Rainforest**	**68.6**	**73.8**	**73.7**	**71.7**	**72.1**	**68.7**
Amazonas	77.5	76.8	71.4	74.5	78.4	74.5
Loreto	79.4	75.5	88.5	81.1	78.3	70.0
Madre de Dios	31.9	31.5	47.0	48.4	53.1	36.7
San Martin	61.6	67.7	68.0	67.9	68.3	66.9
Ucayali	56.7	86.7	60.8	61.6	64.3	70.5

*Source: Reference [30]

**Figure 1:**
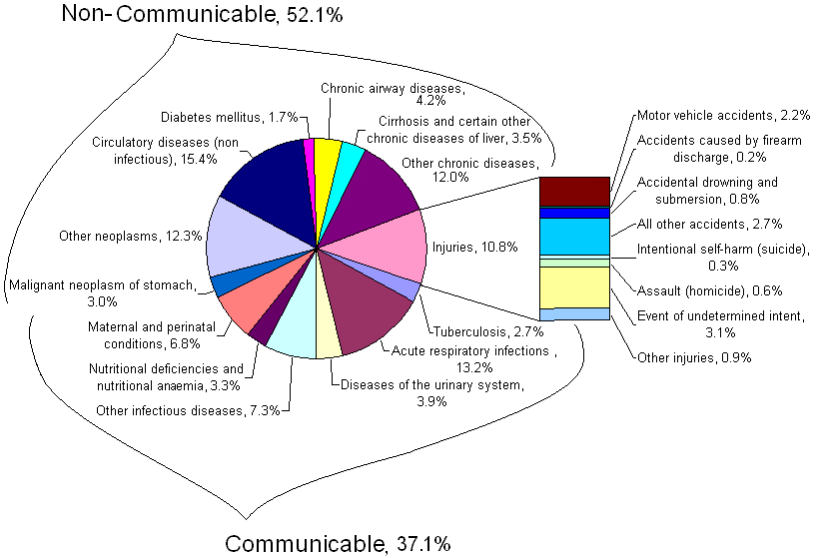
**Main individual causes of death at national level, Peru, period 1996–2000**.

Aggregated national mortality by main causes of death for the five years of study is depicted in Figure 1. For the study period, the group of non-communicable diseases accounts for more than fifty percent of all causes, communicable diseases for more than one third, and injuries for 10.8 percent of all deaths. In the group of 30–69 years old, non-communicable diseases account for 46.2% of all deaths at national level.

**Figure 2:**
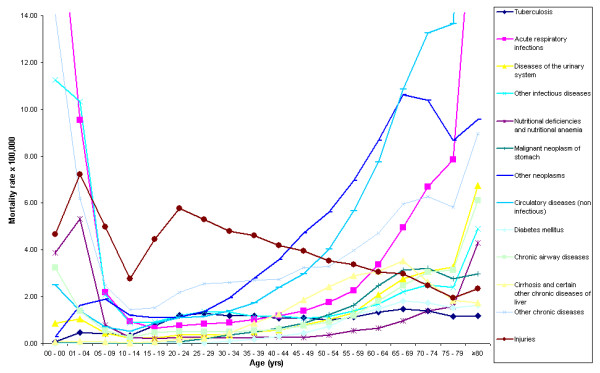
**Thirteen most common causes of death by age group, Peru, period 1996–2000**.

Figure 2 shows the leading causes of death by age groups for the period 1996–2000. It illustrates different death distributions across age groups for the aggregated study period. Four main curve patterns can be observed: bimodal, unimodal, stable, and one of gradual increase. The bimodal pattern is characterized by a peak in each extreme of the curve, with high death rates in young and elderly groups. Acute respiratory infections, nutritional deficiencies and nutritional anaemia are included in this group. Cirrhosis and certain other chronic diseases of liver as well as neoplasms of the stomach display a unimodal curve, showing a gradual increase reaching a peak in the sixth and seventh decades of life, and then a gradual decline. Injuries also show a bimodal distribution with high death rates in childhood and adulthood. As a single cause, injuries constitute the leading cause of death in the group of 5 to 44 years-old. Tuberculosis is a clear example of a stable pattern, with a similar death rate across all ages. Other conditions such as circulatory diseases, diabetes mellitus, and diseases of the urinary system show, as expected, a gradual increase pattern, with the highest death rates in the older age groups.

### 2. Main causes of death by geographical region

**Figure 3:**
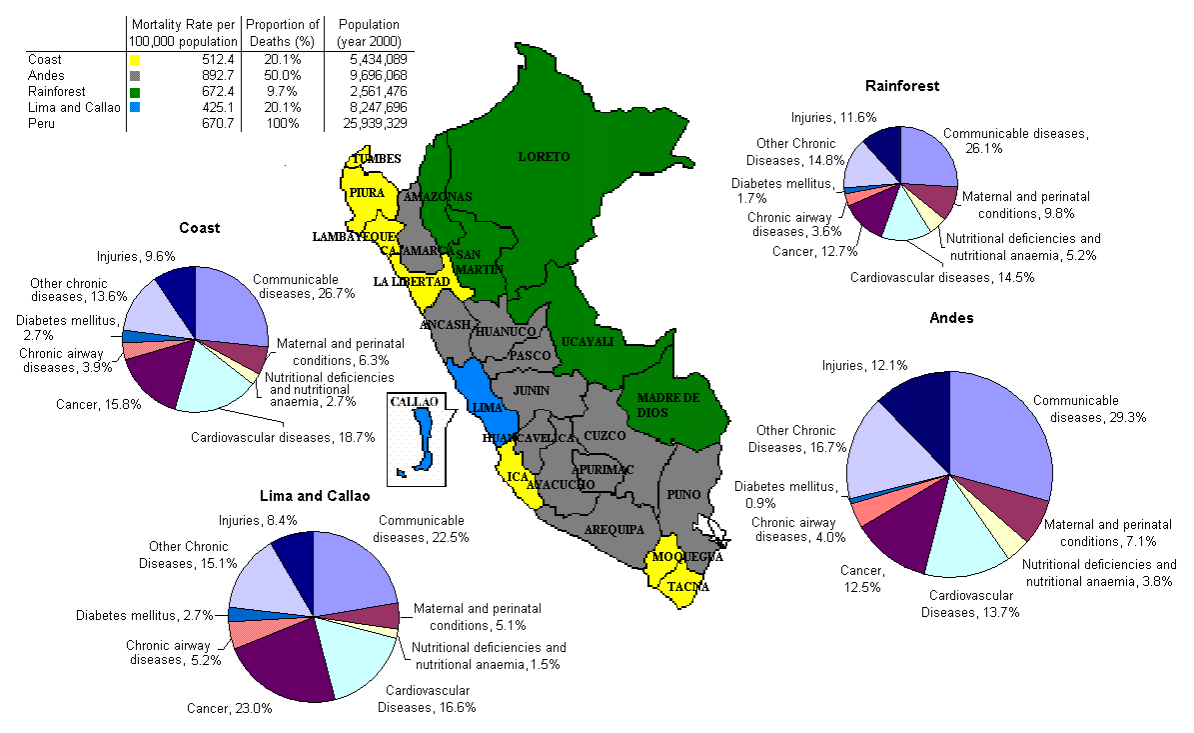
**Regional patterns of mortality for Peru, period 1996–2000 (Size of pies is proportionate to region population)**.

**Figure 4:**
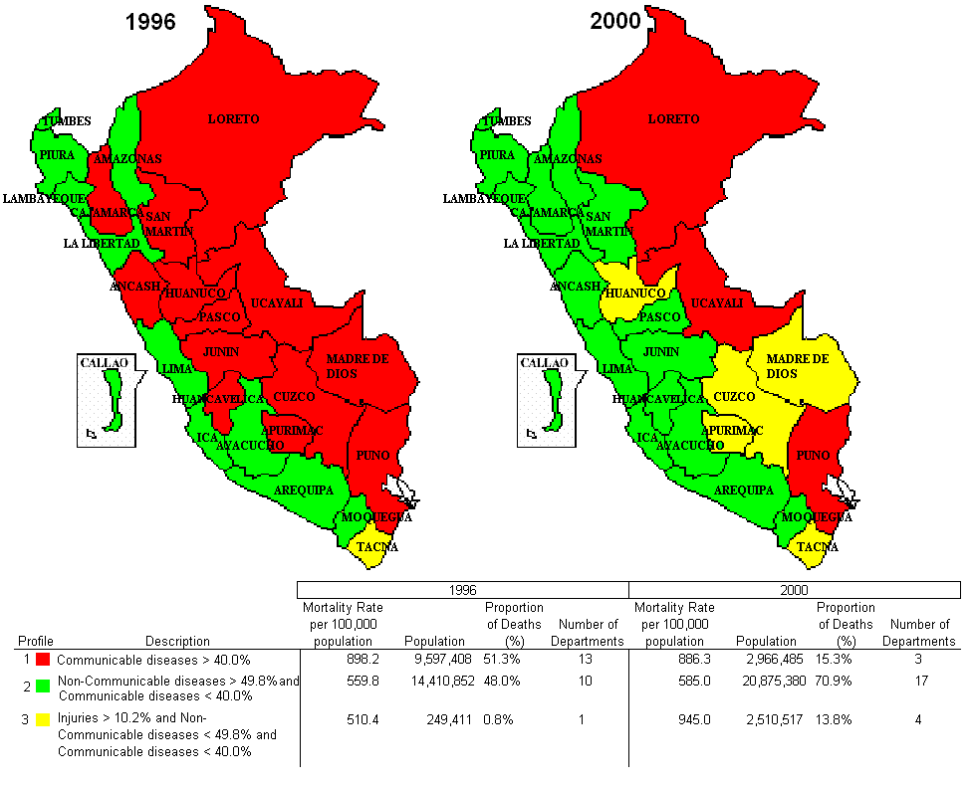
**Mortality profiles for Peru, years 1996 and 2000**.

The proportion of deaths attributable to communicable diseases, injury, and chronic non-communicable diseases such as cardiovascular diseases are high in all regions (Figures 3 and 4). Figure 3 shows the regional patterns of mortality for the study period. In all geographical regions, acute respiratory infections, which comprise mostly pneumonias, are the leading cause of death but show different proportions in each of these areas, ranging from 9.3% in Lima and Callao to 15.3% in the Andean region. As Figure 2 shows, the acute respiratory infections have their greatest impact on the paediatric and the oldest age groups.

### 3. Transition of mortality profiles

Figure 4 shows changes in mortality patterns by department for years 1996 and 2000. In 1996, 11 out of 24 departments had more than 49.8% of their mortality attributed to the group of non-communicable diseases. This increased to 18 departments by the year 2000. In contrast, a sharp decline was observed in the number of departments with deaths dominated by communicable diseases and maternal and perinatal conditions, dropping from 13 to 3 departments by year 2000. The group of injuries showed a substantial increase in the study period, from only one department where it was a prominent cause of death in 1996 to 5 departments in 2000.

### 4. Evolution of mortality time-trends

When national mortality trends for the period of study were analysed (Table 2), a decline was seen in mortality rate per 100,000 population for all ages, from 693.1 in 1996 to 652 in 2000, which corresponds to a cumulative slope of -6.2%. This decline was greater for females (-6.4%) than for males (-5.9%). The same declining trend was also observed in all departments of the country except for Loreto and Puno, which showed marginal increases lower than 1% in cumulative mortality.

**Table 2:** Cumulative slope of registered mortality (%) by regions and by departments, Peru, 1996–2000.

	Infectious diseases		Maternal and perinatal conditions		Nutritional deficiencies and nutritional anaemia		Total
**Coast**	**-8.9%**		**-38.2%**		**-82.0%**		**-6.8%**
Lima and Callao	15.8%		-51.9%		-54.4%		-3.2%
Ica	6.3%		-40.9%		-55.7%		-3.7%
La Libertad	-13.4%		-60.8%		-62.8%		-4.0%
Lambayeque	-7.4%		-18.3%		-80.4%		-5.4%
Moquegua	1.4%		-18.4%		-5.2%		-7.6%
Piura	-11.5%		-37.0%		-105.6%		-9.9%
Tacna	-4.8%		-25.1%		-70.6%		-9.7%
Tumbes	-8.3%		-27.9%		-92.4%		-2.3%
**Andes**	**-23.8%**		**-34.5%**		**-58.6%**		**-6.4%**
Ancash	-9.8%		-31.7%		-152.7%		-8.2%
Apurimac	-18.8%		-52.1%		-72.9%		-10.0%
Arequipa	-15.3%		-42.5%		87.8%		-1.4%
Ayacucho	-14.7%		-8.8%		-73.1%		-6.1%
Cajamarca	-35.6%		-29.3%		-60.8%		-9.3%
Cusco	-27.7%		-50.2%		-71.5%		-10.4%
Huancavelica	-40.9%		-44.5%		-6.4%		-7.1%
Huanuco	-22.7%		-22.7%		-85.9%		-6.9%
Junin	-29.6%		-30.4%		-41.3%		-4.7%
Pasco	-23.5%		-37.9%		-91.9%		-12.5%
Puno	-17.6%		-33.5%		-40.8%		0.6%
**Rainforest**	**-12.0%**		**-38.7%**		**-80.4%**		**-7.3%**
Amazonas	5.2%		-34.6%		-106.0%		-3.6%
Loreto	-1.3%		-33.4%		-44.8%		0.2%
Madre de Dios	-21.9%		-37.5%		-75.9%		-7.1%
San Martin	-35.6%		-46.1%		-130.8%		-18.5%
Ucayali	-14.8%		-43.7%		-77.2%		-9.7%

	Circulatory diseases (non infectious)	Cancer	Chronic airway diseases	Diabetes mellitus	Other chronic diseases	Injuries	Total

**Coast**	**2.2%**	**12.1%**	**-6.8%**	**-17.6%**	**0.6%**	**-10.0%**	**-6.8%**
Lima and Callao	-3.5%	-0.8%	-13.0%	27.6%	-4.5%	-16.5%	-3.2%
Ica	6.3%	5.2%	-25.3%	-15.3%	-2.4%	-12.0%	-3.7%
La Libertad	8.2%	9.7%	3.5%	-1.8%	12.4%	-2.4%	-4.0%
Lambayeque	-6.0%	25.4%	4.6%	-42.9%	-15.1%	-0.6%	-5.4%
Moquegua	-10.5%	-0.6%	-8.4%	-41.5%	-0.4%	-17.1%	-7.6%
Piura	4.0%	9.0%	-4.5%	-13.2%	-3.1%	-15.1%	-9.9%
Tacna	-2.6%	1.8%	-62.9%	-19.7%	25.9%	-26.3%	-9.7%
Tumbes	-4.1%	32.7%	-14.1%	-6.5%	30.1%	-26.0%	-2.3%
**Andes**	**6.5%**	**18.2%**	**17.3%**	**10.3%**	**-0.1%**	**15.3%**	**-6.4%**
Ancash	1.9%	-8.7%	27.8%	-54.8%	5.1%	16.1%	-8.2%
Apurimac	4.3%	16.2%	51.2%	-162.7%	-13.9%	27.1%	-10.0%
Arequipa	-3.5%	11.0%	-1.2%	84.6%	3.5%	-3.8%	-1.4%
Ayacucho	8.1%	10.4%	17.4%	-65.5%	-17.1%	7.4%	-6.1%
Cajamarca	4.5%	30.8%	-15.2%	27.3%	-7.4%	6.6%	-9.3%
Cusco	11.4%	35.4%	30.4%	-3.8%	-17.2%	19.9%	-10.4%
Huancavelica	25.2%	-13.1%	80.0%	56.2%	22.0%	31.5%	-7.1%
Huanuco	-16.6%	17.4%	-11.8%	6.3%	12.1%	27.8%	-6.9%
Junin	2.7%	29.6%	54.2%	25.6%	8.8%	-4.5%	-4.7%
Pasco	-23.5%	15.5%	-41.7%	-8.3%	14.2%	9.5%	-12.5%
Puno	21.6%	34.1%	-20.0%	85.5%	12.7%	29.0%	0.6%
**Rainforest**	**11.1%**	**6.2%**	**-26.4%**	**32.4%**	**10.9%**	**6.6%**	**-7.3%**
Amazonas	12.6%	-15.4%	-53.7%	-65.2%	13.3%	26.8%	-3.6%
Loreto	13.9%	16.3%	-36.2%	76.9%	2.4%	25.5%	0.2%
Madre de Dios	-10.5%	21.5%	60.2%	55.6%	42.4%	-1.0%	-7.1%
San Martin	2.2%	18.1%	-15.6%	4.2%	7.7%	-16.4%	-18.5%
Ucayali	22.8%	-15.1%	64.9%	35.2%	36.6%	1.8%	-9.7%

Table 2 shows the proportion of change for nine death categories by department and by region. Both maternal and perinatal conditions as well as nutritional deficiencies and nutritional anaemia showed marked cumulative declines for the period of study in all departments and all regions. Infectious causes also declined in almost all departments with the exception of four, and in all regions with the exception of Lima. For the rest of conditions the picture is not clearly defined at departmental level, with each department presenting its own peculiarities. For example, Huancavelica and Puno, located in the highland mountainous region of Peru (Andean region), which are among the poorest departments of the country, show consistent increases in mortality in four out of the five conditions related to non-communicable diseases, whereas Amazonas, Lambayeque, Lima and Pasco show a decrease in three non-communicable conditions. These figures should be analysed with caution since minor variations in absolute mortality in a group with a small number of deaths, like diabetes mellitus and chronic airway diseases, could change substantially the cumulative slopes.

When these cumulative changes are analysed by geographical region some findings are noteworthy. The patterns of change for specific groups of causes of death for Lima as a region show a declining pattern, except for diabetes mellitus and infectious diseases (Table 2, Figures 3 and 4). These two latter groups increased by 27.6% and 15.8%, respectively. These changes are closely related to the overall national patterns of change, except for injuries, for which a 16.5% decrease is observed in Lima and a 4.4% increase for Peru's national aggregate. Circulatory diseases and cancer show an increase in all regions except Lima and the national level. Diabetes mellitus presents a reduction only in the coastal region, with an increasing pattern in the remaining three regions. Injuries also show a reduction in the coastal region, as well as in Lima. The changes observed in injuries did not follow a uniform pattern, showing a decrease of 16.5% in Lima region, and an increase of 15% in the Andean region.

## Discussion

According to the World Health Organization, it is estimated that about 35 million deaths due to chronic diseases occur annually at global level [40]. The main components of these deaths are heart disease, stroke, cancer, and other chronic diseases. Eighty percent of these deaths occur in low-income and middle-income countries, mainly among adults aged 30–69 years, with equal numbers among men and women [10]. Emphasis on preventive and control measures at international level has the potential of preventing 36 million deaths by 2015 [10].

Peruvian national mortality by all causes and by age and sex decreased across the period 1996–2000 by 6.2%. A marked disparity in overall mortality exists between Lima as a region and the Andes. While the former accounts for 32% of total population, it only represented 20% of total deaths in our study. The Andean region, which represents 38% of Peru's population, accounted for half of all country deaths.

The elaboration of mortality profiles for each department using 1996 as a baseline show a clear shift in the predominance of causes of death from communicable to non-communicable diseases. Of note, non-communicable diseases accounted for 46.2% of all deaths in the age group of 30–69 years, a figure that is in accordance with international reports [10,40]. The study of cumulative mortality by changes in slope during the study period provides also valuable insights to the understanding of mortality patterns within the country.

Heterogeneity of causes of mortality was observed when the analyses were performed by departments and regions. This heterogeneity is observed even within the major groups of deaths. For example, while cerebrovascular disease is a common cause of death in all regions in Peru, ischemic heart disease is prominent only in Lima. Such pattern coincides with the description of the epidemiological transition made specifically for cardiovascular diseases [41], whereby different conditions within this group are more or less prominent in sub-national settings facing different stages of the transition.

A surprising finding was that injuries decreased by 16.5% in Lima and by 10% in the coast from the period 1996 through 2000, but it increased by 15.3% in the Andean region in the same period. These changes are potentially cancelled out when using national single aggregated data. It is clear, conversely, that mortality due to maternal and perinatal conditions, as well as those related to nutritional deficiencies; show an important and consistent decrease in all regions and departments during the study period. This was not the case for mortality due to infectious diseases.

Such information, disaggregated by within-country regions, will provide a valuable resource to address the main areas deserving particular attention from the policy level. Interpreting and understanding the epidemiological transition described for Peru, beyond historical approaches [24], requires careful study. The marked reduction in the group of communicable diseases observed in several departments does not mean that this group no longer constitutes a major health problem for Peru. The message is, instead, that Peru has areas with a predominance of both communicable and non-communicable diseases. Epidemiological transition characterized by different patterns of mortality is more frequently described for different world regions in international reports, with less emphasis on within country differences [39]. Further sub-national level studies are needed in developing transitional countries like Peru to identify specific differing patterns of mortality at local level.

Hence, Peru appears to face a double burden of communicable and non-communicable diseases, and the same time, the increase of injuries as a major cause of death. This scenario poses a formidable challenge for defining health policy priorities and also for shaping the structure and dynamics of the health system at national and local levels, if we are going to deal successfully with such changes. Thus, the main lesson to learn is that national and local health policies need to be redefined, taking into account the changing pattern of deaths across the country. This will require the adaptation of national and local health systems, which until now have been conceived to face almost exclusively communicable diseases. Additionally, the rapid changes in the population structure pose huge challenges to the whole health system, encompassing the broad range of policy making, community empowerment, accountability, financial resources, budget allocation, human health resources, as well as pension fund systems.

Standard methods developed and adopted by international agencies (PAHO and CELADE) have been followed in this report, to ensure the best possible analytical approach, although no correction method can replace a robust vital registration data source. Various direct and indirect methods for assessing the completeness of vital registration systems have been developed [42] Basically, the direct methods use independently constructed listing of deaths, whereas the indirect methods resort to other data on the population, particularly its age distribution or growth rate. The "capture-recapture" approach assesses directly completeness, through comparison of deaths reported in an independent mortality survey with the deaths reported by the registration systems for the same population. This approach allows the identification and estimation of unmatched and unrecorded deaths, but it needs a minimum population sample size for being meaningful, which may increase substantially the cost. The indirect demographic methods for estimating the completeness of the registered death data include, among others, the Brass Balance method, the Bennet-Horiuchi method, the Preston and Hill method, and the CELADE method. Each of these indirect methods makes use of the age distribution of reported deaths within a certain age range, which is compared with the age distribution of the population. Each of them also assumes that the population is stable and closed to migration. In addition, they can use various combinations of data and assumptions, including data on the age distribution of the population in one or two points in time, assumption of population stability (that the population is characterized by constant mortality and exponential growth in the annual number of births), and assumption that registered deaths represent a constant proportion of true deaths at each age. A detailed description of the direct and indirect methods and of their advantages and limitations can be found in a review by Preston [42].

Despite the advantages and strengths outlined, it is also important to acknowledge some limitations of this study. It has been correctly argued that Peru's registered mortality data is of low quality [32]. Completeness and coverage of the information are far from optimal at departmental level. Timeliness of information reporting is affected by the scarcity of qualified human resources and financial support, and by lack of efficient accountability mechanisms. The causes of death in our study were based on clinical diagnosis prior to death and on medical autopsy. Misclassification and erroneous attribution of causes of deaths are intrinsic problems with these types of data and they need to be addressed. Although we found that the proportion of garbage codes was still substantially high, ranging from 12 to 24 percent during the study period, we were not able to assess systematically the quality of attribution of causes of death in this report, and have assumed largely that each recorded death was actually due to that cause. These "garbage codes" include cardiovascular disease categories without diagnostic meaning, such as cardiac arrest and heart failure, deaths where the intent was not determined, and cancer deaths coded to categories for secondary or unspecified sites. Also, two different coding systems for deaths, ICD-9 and ICD-10, were in use in Peru during the study period. Despite the use of guidelines for the appropriate matching of codes [35], it is recognised that this process is not perfect and some deaths will end up contributing to different groups of deaths depending on the year of study. This bias could be expected to be more pronounced if individual causes of deaths were analysed but not in our case, where groups of cases were the units of analysis. This bias is present, however, in the case of "cardiac diseases" which ranked among the first three leading causes of death in all regions. This group, due to the coding system used, includes cardiac arrest – a common cause of death prone to error in cause attribution and over ascertainment – and heart disease. Additionally, the life table that we used for estimating the under-registration of deaths during the study period had the same demographic parameters for the whole period, thus introducing a potential bias, although changes in population structure during the period under study might have been minor. Finally, a longer study period would have been better to assess more accurately mortality time-trends, but this was not feasible with the available data.

While efforts are in progress for improving quality of certification and coding of causes of death at health services in Peru, we could not identify a Peruvian comprehensive audit system currently in place, and we are not aware of any published study assessing systematically the above noted problems in this country, to suggest implementation of specific solutions.

Thus we must acknowledge that these data present substantial problems to address mortality due to under-registration and cause-attribution, but it is the best available evidence on mortality for Peru. Alternative sources of information such as the Demographic and Health Surveys are of importance and widely used, but the mortality data generated are related to the five to ten years before the survey. This provides an additional reason for making the effort to study mortality data available from vital registration systems, while maintaining a critical approach on their accuracy and completeness.

## Conclusion

Our paper contributes to understand the importance of assessing disaggregated mortality data within a country. This information will, in turn, allow policy makers to identify public health priorities and implement the most appropriate interventions. The analysis of trends will also contribute to understand changes observed in different settings, and moreover, can inform the adoption of early interventions to counteract unfavourable trends of mortality in their early stages within a particular setting. Current patterns of changes in diabetes mellitus and injuries deaths raise for instance important questions and serve as warning signals, particularly for some areas within the country.

## Competing interests

The authors declare that they have no competing interests.

## Authors' contributions

LH, MT, JJM and WM conceived the study and its design. LH, MT and FG took part in data management and analysis. LH and JJM wrote the first draft of the report. All authors contributed to interpretation of the data and writing of the report. All authors read and approved the final manuscript. The corresponding author had full access to all the data in the study and had final responsibility for the decision to submit for publication.

## Pre-publication history

The pre-publication history for this paper can be accessed here:



## Supplementary Material

Additional File 1 **Total Number and proportion of deaths assigned to 'Signs, Symptoms and Ill defined conditions' (SSIDC) for the uncorrected data, by age group and by year.** The data describe in detail the yearly total number and proportion of deaths assigned signs, symptoms and ill defined conditions for the uncorrected data, by age group and by year, for the period 1996–2000 Click here for file

Additional file 2 **Abridged CELADE life table for Peru, Period 1995–2000.** The data provided present summary information including the age in (years), central rate, probability of dying, deaths, stable population; time lived and life expectancy by age and sex for the period 1995–2000. Click here for file

Additional File 3 **Standardized death rate and population by department and by year. **This file represents standardized death rate and population by department and region, and by year for the period 1996–2000. Click here for file

## References

[B1] Moser K, Shkolnikov V, Leon DA (2005). World mortality 1950–2000: divergence replaces convergence from the late 1980s. Bull World Health Organ.

[B2] Lopez AD, Mathers CD, Ezzati M, Jamison DT, Murray CJ (2006). Global and regional burden of disease and risk factors, 2001: systematic analysis of population health data. Lancet.

[B3] Lopez AD, Mathers CD, Ezzati M, Jamison DT, Murray CJL (2006). Global burden of disease and risk factors.

[B4] Bloom B, Michaud C, La Montagne J, Simonsen L, Jamison DT, Breman JG, Measham AR, et al (2006). Priorities for Global Research and Development of Interventions. Disease Control Priorities in Developing Countries.

[B5] World Health Organization (2006). World Health Statistics 2006.

[B6] Murray CJ, Kulkarni SC, Michaud C, Tomijima N, Bulzacchelli MT, Iandiorio TJ, Ezzati M (2006). Eight Americas: Investigating Mortality Disparities across Races, Counties, and Race-Counties in the United States. PLoS Medicine.

[B7] Boerma JT, Stansfield SK (2007). Health statistics now: are we making the right investments?. Lancet.

[B8] Miranda JJ, Patel V (2005). Achieving the Millennium Development Goals: Does Mental Health Play a Role?. PLoS Medicine.

[B9] Perel P, Casas JP, Ortiz Z, Miranda JJ (2006). Noncommunicable Diseases and Injuries in Latin America and the Caribbean: Time for Action. PLoS Medicine.

[B10] World Health Organization (2005). Preventing chronic diseases: A vital investment. WHO Global Report.

[B11] Wallace RB (2001). Bridging epidemiology and demography: theories and themes. Ann N Y Acad Sci.

[B12] Omran AR (1971). The epidemiologic transition. A theory of the epidemiology of population change. Milbank Mem Fund Q.

[B13] Popkin BM (2003). Dynamics of the nutrition transition and its implications for the developing world. Forum Nutr.

[B14] Popkin BM (2002). The shift in stages of the nutrition transition in the developing world differs from past experiences!. Public Health Nutr.

[B15] Frenk J, Bobadilla JL, Stern C, Frejka T, Lozano R (1991). Elements for a theory of the health transition. Health Transit Rev.

[B16] Wang L, Kong L, Wu F, Bai Y, Burton R (2005). Preventing chronic diseases in China. Lancet.

[B17] Yang G, Kong L, Zhao W, Wan X, Zhai Y, Chen LC, Koplan JP Emergence of chronic non-communicable diseases in China. Lancet.

[B18] Joshi R, Cardona M, Iyengar S, Sukumar A, Raju CR, Raju KR, Raju K, Reddy KS, Lopez A, Neal B (2006). Chronic diseases now a leading cause of death in rural India – mortality data from the Andhra Pradesh Rural Health Initiative. Int J Epidemiol.

[B19] Albala C, Vio F (1995). Epidemiological transition in Latin America: the case of Chile. Public Health.

[B20] Albala C, Vio F, Yanez M (1997). Epidemiological transition in Latin America: a comparison of four countries. Rev Med Chil.

[B21] Fraser B, Zuniga A, Parada I (2005). Health care costs and financial consequences of epidemiological changes in chronic diseases in Latin America: evidence from Mexico. Public Health.

[B22] Frenk J, Frejka T, Bobadilla JL, Stern C, Lozano R, Sepúlveda J, José M (1991). [The epidemiologic transition in Latin America]. Bol Oficina Sanit Panam.

[B23] Fraser B (2006). Peru's epidemiological transition. Lancet.

[B24] Huynen MM, Vollebregt L, Martens P, Benavides BM (2005). The epidemiologic transition in Peru. Rev Panam Salud Publica.

[B25] World Bank Data & Statistics: Country Groups. http://web.worldbank.org/WBSITE/EXTERNAL/DATASTATISTICS/0,,contentMDK:20421402~pagePK:64133150~piPK:64133175~theSitePK:239419,00.html#Lower_middle_income.

[B26] Instituto Nacional de Estadística e Informática (2006). Resultados Definitivos. Censo 2005. Censo 1993 Lima.

[B27] World Bank World Development Indicators database. GNI per capita 2005, Atlas method and PPP.

[B28] World Health Organization (2006). The World Health Report 2006 – Working together for health. World Health Organization, Geneva.

[B29] United Nations Development Programme (2000). Human Development Report 2000.

[B30] ENAHO (2001). Instituto Nacional de Estadistica e Informatica 1996–2000. Encuesta Nacional de Hogares, ENAHO, 1996–2000. Instituto Nacional de Estadistica e Informática.

[B31] Valdivia M (2002). Acerca de la magnitud de la inequidad en salud en el Perú. Lima, GRADE.

[B32] Mathers CD, Fat DM, Inoue M, Rao C, Lopez AD (2005). Counting the dead and what they died from: an assessment of the global status of cause of death data. Bull World Health Organ.

[B33] Huicho L, Trelles M, Gonzales F (2006). National and sub-national under-five mortality profiles in Peru: a basis for informed policy decisions. BMC Public Health.

[B34] Pan American Health Organization Technical Notes. Health statistics from the Americas. 2006 ed.

[B35] Anonymus (1999). Norms and Standards in Epidemiology: New PAHO list 6/67 for tabulation of ICD-10 mortality data. Epidemiol Bull.

[B36] Organización Panamericana de la Salud (1992). Estadísticas de Salud de las Américas. Publicación Científica 542.

[B37] Silvi J (2003). On the estimation of mortality rates for countries of the Americas. Epidemiol Bull.

[B38] Instituto Nacional de Estadística e Informática and Macro International (2000). Encuesta Demográfica y de Salud Familiar 2000. Lima, Perú INEI and Macro International.

[B39] Jamison DT, Breman JG, Measham AR, Alleyne G, Claeson M (2006). Disease Control Priorities in Developing Countries.

[B40] Strong K, Mathers C, Leeder S, Beaglehole R (2005). Preventing chronic diseases: how many lives can we save?. Lancet.

[B41] Yusuf S, Reddy S, Ounpuu S, Anand S (2001). Global burden of cardiovascular diseases: Part I: general considerations, the epidemiologic transition, risk factors, and impact of urbanization. Circulation.

[B42] Preston SH (1981). Use of Direct and Indirect techniques for estimating the completeness of registration systems. Data bases for mortality measurement. Proceedings of the United Nations/World Health Organization Working Group on Data Bases for Measurement of Levels, Trends, and Differentials in Mortality; Bangkok.

